# Intussusception in Infants

**Published:** 1901-11-09

**Authors:** 


### INTUSSUSCEPTION IN INFANTS.

At the last meeting of the Clinical Society an interesting question arose as to the method of operating for intussusception in infants. Mr. Wallis showed the case—one of acute intussusception in a child aged seven months, successfully treated by operation. The operation was performed 33 hours after the first onset of the symptoms. The abdomen was opened by an incision in the upper part of the right semilunar line, and the intussusception, which occupied the situation of the hepatic flexure of the colon, was drawn out of the incision. By gentle traction and pressure it was gradually reduced. The intussuscepted portion was seven inches long. The operation lasted 20 minutes. The bowels acted the next day, and the child recovered. In discussing this case Mr. Barker asked for more particulars of the operation, for he considered that it was of great importance in such cases that only a small opening into the abdomen should be made, and that the gut, instead of being brought outside, should be manipulated with the first finger in the abdomen and the thumb on the abdominal wall. Mr. Wallis, in reply, stated that in this case it had been impossible to reduce the intussusception within the abdomen, and that it had therefore been brought outside, when it was easily reduced. There can, of course, be no doubt that Mr. Barker is right—if his contention is that those cases do the best in which it is possible to obtain reduction by the manipulation which he described. But, equally of course, when such means fail further measures must be undertaken.

